# Diversity and Biogeography of Human Oral Saliva Microbial Communities Revealed by the Earth Microbiome Project

**DOI:** 10.3389/fmicb.2022.931065

**Published:** 2022-06-13

**Authors:** Jinlan Wang, Jianqing Feng, Yongbao Zhu, Dandan Li, Jianing Wang, Weiwei Chi

**Affiliations:** ^1^National Administration of Health Data, Jinan, China; ^2^96608 Army Hospital of Chinese People’s Liberation Army, Hanzhong, China; ^3^State Key Laboratory of Microbial Technology, Institute of Microbial Technology, Shandong University, Qingdao, China

**Keywords:** oral cavity, saliva, microbiome, earth microbiome project, microbial diversity, environmental microbiome

## Abstract

The oral cavity is an important window for microbial communication between the environment and the human body. The oral microbiome plays an important role in human health. However, compared to the gut microbiome, the oral microbiome has been poorly explored. Here, we analyzed 404 datasets from human oral saliva samples published by the Earth Microbiome Project (EMP) and compared them with 815 samples from the human gut, nose/pharynx, and skin. The diversity of the human saliva microbiome varied significantly among individuals, and the community compositions were complex and diverse. The saliva microbiome showed the lowest species diversity among the four environment types. Human oral habitats shared a small core bacterial community containing only 14 operational taxonomic units (OTUs) under 5 phyla, which occupied over 75% of the sequence abundance. For the four habitats, the core taxa of the saliva microbiome had the greatest impact on saliva habitats than other habitats and were mostly unique. In addition, the saliva microbiome showed significant differences in the populations of different regions, which may be determined by the living environment and lifestyle/dietary habits. Finally, the correlation analysis showed high similarity between the saliva microbiome and the microbiomes of Aerosol (non-saline) and Surface (non-saline), i.e., two environment types closely related to human, suggesting that contact and shared environment being the driving factors of microbial transmission. Together, these findings expand our understanding of human oral diversity and biogeography.

## Introduction

The oral cavity is an important place for the delivery and exchange of substances inside and outside the human body and is also a gateway for pathogens and toxic substances to invade the body. The microbes found in the human oral cavity are collectively referred to as the oral microbiome (Gao et al., 2018; Mark Welch et al., 2019, 2020). The oral cavity connects the external environment with the digestive tract and respiratory tract, and the complex and variable interaction of oral microbes helps the body fight against undesirable external stimuli. Imbalances in the microbial community can lead to oral diseases such as dental caries, periodontitis, oral mucosal diseases, and even some other diseases, such as autoimmune diseases, cardiovascular diseases, diabetes, cancers and neurodegenerative disorders (Jorth et al., 2014; Atarashi et al., 2017; Peters et al., 2017; Blod et al., 2018; Gao et al., 2018; Lira-Junior and Bostrom, 2018; Philip et al., 2018; Plaza-Diaz et al., 2018; Reddy et al., 2018; Saikaly et al., 2018; Wasfi et al., 2018; Bacali et al., 2022). Therefore, the oral microbiome plays an important role in maintaining the balance between human microbial communities and human health, and also in the onset and progression of several localized and systemic diseases including those of bacterial, viral and fungal origin (Zarco et al., 2012; He et al., 2015; Soffritti et al., 2021). However, compared to the gut microbiome, the oral microbiome has received little attention.

There are multiple microenvironments in the oral cavity that communicate with each other through saliva. The composition of the oral microbiome is complex, and the expanded Human Oral Microbiome Database (eHOMD) includes 770 microbial species of 230 genera in 16 bacterial and archaeal phyla (Escapa et al., 2018). Of all the species in this database, 57% are officially named, 13% are unnamed but cultivated and 30% are known only as uncultivated phylotypes. There is no difference among the oral, gut, and skin microbiomes of newborn babies, but the composition of their oral microbiomes will change significantly as age increases and dentition changes (Dominguez-Bello et al., 2010). The differences in the oral microbiomes at different time points for the same individual are significantly lower than those in the gut, skin and other body parts (Costello et al., 2009). The effects of the early living environment on shaping oral microbes are much greater than those of genetic factors (Shaw et al., 2017). In addition, lifestyle habits, social factors, and oral pH value also affect the composition of the oral microbiome (Willis et al., 2018).

The Earth Microbiome Project (EMP) aims to collect as many of the Earth’s microbial communities as possible to promote our understanding of the relationship between microbes and the environment, including plants, animals and humans (Gilbert et al., 2010, 2014). The first data published by EMP contained 27,751 samples from 97 independent studies representing different environmental types, geographic locations, and chemical reactions (Thompson et al., 2017). All samples were subjected to DNA extraction and sequencing, and the bacterial and archaeal parts of the entire database were analyzed. Here, using sequencing data from 404 human oral saliva samples published by EMP, we explored the characteristics, core taxa of human oral microbiome and their association with the environmental microbiome, comparative analysis among them with human gut, nose/pharynx and skin microbes.

## Results and Discussion

### Prokaryotic Composition in the Human Oral Saliva Habitat

We analyzed the sequenced data of 404 human oral saliva samples from 5 independent studies (Supplementary Data). For further calculation, 5000 observed sequences were randomly extracted from each sample. All samples were subjected to the Deblur algorithm to remove erroneous sequences and to calculate operational taxonomic units (OTUs) at single nucleotide precision.

The results showed that the average number of observed bacterial and archaeal OTUs was 71.25 ± 26.40 in human oral samples, with a maximum of 216 OTUs and a minimum of 25 OTUs in a single sample. The Chao1 index is relatively sensitive to low-abundance species. The average Chao1 index for human oral samples was 85.83 ± 34.12, ranging from 28.75 to 258.00. The Shannon index can simultaneously reflect species diversity and community uniformity. The average Shannon index for human oral samples was 3.61 ± 0.76, varying from 0.97 to 5.34. Faith’s PD value (Faith’s phylogenetic diversity) is a good measure of phylogenetic diversity, and the average of Faith’s PD value for human oral samples was 11.87 ± 2.90, varying between 6.03 and 29.84. These results indicated that the diversity of the human oral microbiome was significantly different among individuals.

**FIGURE 1:**
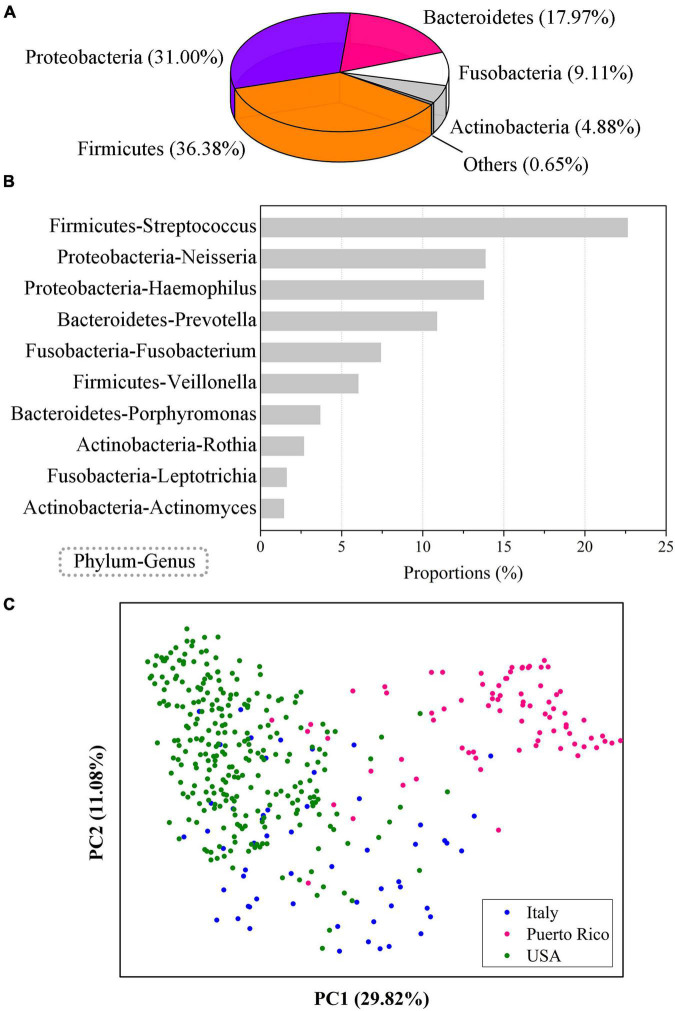
Community composition of the human oral saliva microbiome. **(A)** Community composition of the human saliva microbiome at the phylum level. **(B)** Ten human saliva microbial genera with abundances over 1%. **(C)** PCoA analysis of the oral microbes from populations in different regions. The results were computed based on 404 EMP saliva samples from 3 regions, including 56 in Italy, 79 in Puerto Rico, and 269 in the United States.

The predominant phyla of the human oral saliva microbiome were *Firmicutes*, *Proteobacteria*, *Bacteroidetes*, *Fusobacteria*, and *Actinobacteria*, with average relative abundances of 36.38, 31.00, 17.97, 9.11, and 4.88%, respectively (Figure 1A). The total relative abundance of the 10 predominant genera (>1%) was 83.88%, and *Streptococcus* (22.62%), *Neisseria* (13.86%), and *Haemophilus* (13.76%) were the top three genera in terms of average relative abundance (Figure 1B). These 10 high-abundance genera in human oral samples were distributed in multiple bacterial phyla, of which *Firmicutes*, *Proteobacteria*, *Bacteroidetes*, *Fusobacterium*, and *Actinomycetes* each contained two genera. Therefore, the human oral microbial communities show high complexity in community composition.

The human oral microbial samples were obtained from 3 regions, including 56 in Italy, 79 in Puerto Rico, and 269 in the United States. We found that the diversity of the oral microbiome was significantly different (Analysis of Variance, ANOVA, *p* < 0.01) among the populations of these four regions, and there were also obvious differences in community composition. A principal coordinate analysis (PCoA) based on Bray-Curtis distance showed that oral samples from the three regions were clearly distinguishable (Figure 1C). Therefore, although the individual differences in human oral microbiota were obvious, significant regional differences could still be observed, which might be related to the differences in living environment, dietary habits and other factors of populations in different regions.

### Comparative Analyses of Prokaryotic Biodiversity Among Four Human Habitats

In addition to the oral cavity, the gut, nose/pharynx and skin are also important habitats for human microbial colonization. Using the data published by EMP, we compared the differences between the human oral microbiome and the gut, nasal/pharyngeal, and skin microbiomes. Among them, gut microbial data were from 216 samples, nasal/pharyngeal data were from 253 samples, and skin data were from 346 samples.

**FIGURE 2:**
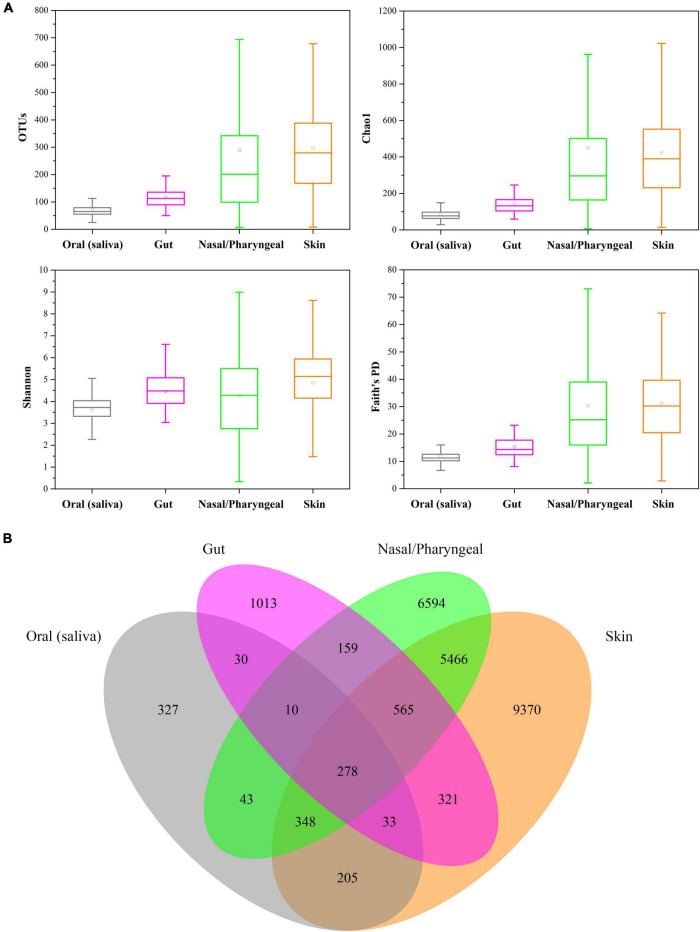
Comparisons of the diversity of human oral saliva, gut, nasal/pharyngeal and skin microbiomes. **(A)** Comparison of alpha diversity, with diversity calculated sequentially as observed OTU, Shannon index, Chao1 index, and Faith’s PD value. **(B)** Number of unique and cross-habitat distributed OTUs. Analysis was performed based on 216 gut, 253 nasal/pharyngeal and 346 skin samples from the EMP.

The results showed that the human oral microbiome diversity was significantly (ANOVA, *p* < 0.01) lower than that of the gut, nasal/pharyngeal, and skin microbiomes (Figure 2A). The average number of observed bacterial and archaeal OTUs was 117 ± 40 in human gut samples, 289 ± 285 in human nasal/pharyngeal samples, and 297 ± 177 in human skin samples, each of which was significantly higher than the average value observed in human oral samples. The average values of the Chao1 index for human gut, nasal/pharyngeal and skin samples were 140.29 ± 49.75, 449.81 ± 469.85, and 422.42 ± 271.39, respectively, which were significantly higher than 85.83 ± 34.12 for oral samples. The average Shannon indexes for human gut, nasal/pharyngeal and skin samples were 4.45 ± 0.81, 4.27 ± 2.00, and 4.85 ± 1.60, respectively, which were significantly higher than 3.61 ± 0.76 for the oral sample. In addition, the average Faith’s PD values for human gut, nasal/pharyngeal and skin samples were 15.30 ± 4.35, 30.26 ± 21.22, and 31.30 ± 14.53, respectively, which were also significantly higher than 11.87 ± 2.90 for oral samples.

Although the oral saliva habitat contained the most samples, only 1274 OTUs were detected in all 404 samples, which was much lower than 2409 OTUs in gut samples, 13,463 OTUs in nasal/pharyngeal samples and 16,586 OTUs in skin samples, indicating that the low diversity of the human oral saliva microbiome once again (Figure 2B). Moreover, for these four habitats, only 25.67% of the OTUs in oral saliva habitats were unique to it and did not exist in the other three types of habitats, while the corresponding values of human gut, nasal/pharyngeal and skin habitats were 42.05, 48.98, and 56.50%, respectively. Notably, 278 OTUs could be detected in all four types of habitats, accounting for 21.82% of all OTUs in the oral cavity.

### Prokaryotic Composition Differences Among Four Human Habitats

**FIGURE 3:**
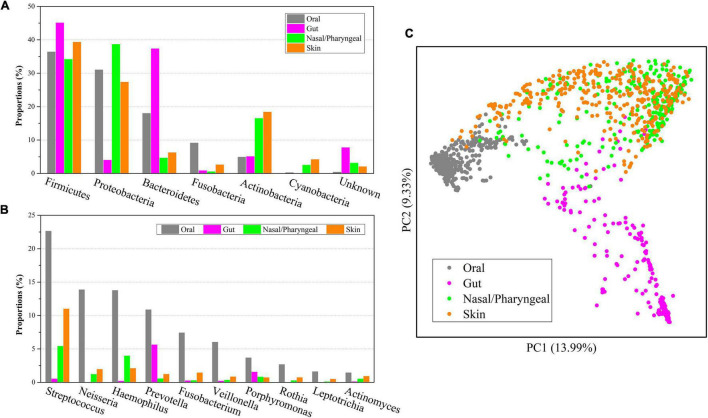
Composition comparisons of human oral saliva, gut, nasal/pharyngeal and skin microbiomes. Distributions of six microbial phyla with the highest abundance **(A)** and 10 genera with the highest abundance **(B)** of the oral microbiome in other body parts. PCoA analysis **(C)** of microbial composition from four different parts of the human oral saliva, gut, nose/pharynx and skin.

*Firmicutes* was not only the most abundant microbe at the phylum level in the oral microbiome but also had more than 30% abundance in other body locations, and its abundance in the gut microbiome was as high as 45.06% (Figure 3A). *Proteobacteria* had an abundance of more than 25% in the oral, nasal/pharyngeal and skin microbiomes but only 3.98% in the gut microbiome. *Bacteroidetes* accounted for 17.97% in the oral microbiome and 37.33% in the gut microbiome but only 4.64% and 6.22% in the nasal/pharyngeal and skin microbiomes, respectively. The abundance of *Fusobacteria* in the oral (9.11%) microbiome was significantly higher than that in the gut (0.84%), nasal/pharyngeal (0.52%) and skin (2.58%) microbiomes. The abundance of *Actinobacteria* in the oral (4.88%) microbiome was close to that of the gut (5.06%) microbiome but significantly lower than that of the nasal/pharyngeal (16.47%) and skin (18.38%) microbiomes. The abundance of *Cyanobacteria* in the oral (0.24%) microbiome was higher than that in the gut (0.02%) microbiome but lower than that in the nasal/pharyngeal (2.49%) and skin (4.17%) microbiomes. For the 10 genera with more than 1% abundance in the oral microbiome, their abundances in the gut, nasal/pharyngeal and skin microbiomes were significantly lower than those in the oral cavity (Figure 3B). For example, the abundance of *Neisseria* in the oral cavity was 13.86%, while the abundances in the gut, nasal/pharyngeal and skin microbiomes were only 0.003, 1.20, and 1.94%, respectively.

Furthermore, we performed a PCoA analysis based on the Bray-Curtis distance for 1,219 samples from the human oral cavity, gut, nose/pharynx, and skin, and displayed them in a scatter plot (Figure 3C). The results showed that the microbiome of oral samples could be well distinguished from the microbiomes of other body part samples, indicating that the oral microbiome was significantly different from other parts in community composition. Similarly, the microbiome for gut samples could also be well distinguished from the microbiomes of other body part samples. However, there was considerable overlap for the microbiomes between the nasal/pharyngeal and skin samples. The lowest dispersion of the oral microbiome among the four microbiomes suggested the lowest diversity, which was consistent with the alpha diversity index. The clustering analysis indicated that the nasal/pharyngeal and skin microbiomes were most similar, while the oral microbiome was more similar to the nasal/pharyngeal and skin microbiomes relative to the gut microbiome.

### Core Operational Taxonomic Units of the Human Oral Saliva Microbial Communities

**FIGURE 4:**
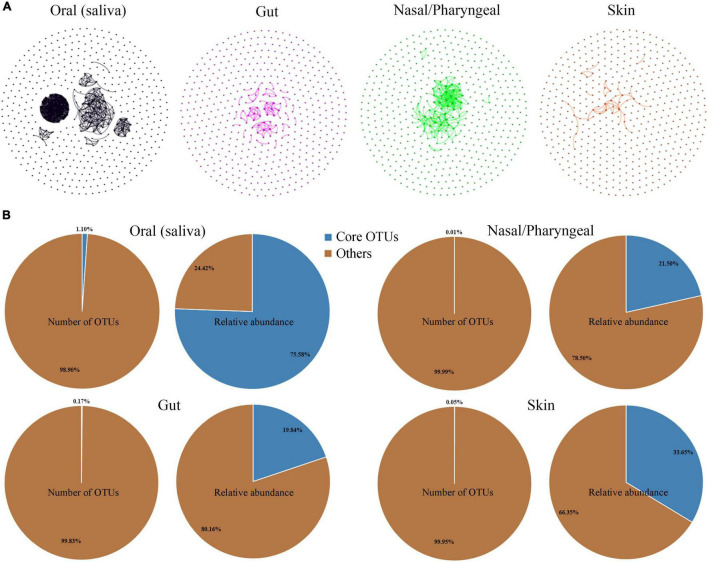
Comparison of the dominant taxa and core taxa in the four habitats of human oral saliva, gut, nose/pharynx, and skin. **(A)** Co-occurrence network analysis of the dominant bacterial OTUs (top 500) of the different habitats. Each node represents an OTU, and the line indicates a significant correlation between the two OTUs (Pearson test, *r* > 0.5, *p* < 0.05). **(B)** Proportions of OTU number and abundance of core taxa in the four habitats of human oral saliva, gut, nose/pharynx, and skin.

Most microorganisms do not live in isolation; they thrive in communities with large numbers and develop close interactions that generate increased benefits for the group. Network inference techniques have frequently been applied to microbial interactions (Faust and Raes, 2012). To analyze the degree of interactions among dominant microbial taxa in different habitats, OTUs with the top 500 abundance were selected from gut, nasal/pharyngeal, oral saliva and skin samples to construct a co-occurrence network, respectively. The total relative abundance of these OTUs reached 97.42, 91.96, 99.74, and 90.52% in gut, nasal/pharyngeal, oral and skin samples, respectively, suggesting that they occupy the majority of the microbial community. The results showed that the aggregation of the microbial community network was significantly different among different habitats, indicating that there were significant differences in the interaction degree of dominant microbial taxa (Figure 4A). Specifically, oral showed the highest degree of network aggregation among the four habitats, followed by nose/pharynx, gut and skin. The parameters representing the correlation-based network topological structures were calculated; these parameters also showed that the edge and average degree were highest in the oral cavity, followed by the nose/pharynx, gut and skin.

We identified core taxa of prokaryotes in the habitat based on the criteria that they were present in at least 80% of the samples and had a total abundance of not less than 1% in all samples. The results showed that the core taxa of the human oral habitat contained only 14 OTUs, accounting for 1.10% of all oral OTUs, but with a total sequence abundance of 75.58% (Figure 4B). The origins of these core taxa members were extensive, with 6 OTUs classified as *Firmicutes*, 4 in *Bacteroidetes*, 2 in *Proteobacteria*, 1 in *Fusobacteria*, and 1 in *Actinobacteria*. Similarly, we also analyzed gut, nasal/pharyngeal, and skin habitats, but only identified 4, 2, and 8 core OTUs, respectively. These core taxa also had a very low proportion of OTUs (0.17% in gut, 0.01% in nose/pharynx, and 0.05% in skin) but occupied a high sequence abundance (19.84% in gut, 21.50% in nose/pharynx, and 33.65% in skin). Core taxa had the greatest impact on the oral habitat among the four habitats, and most of these core taxa were unique to the oral habitat. The core taxa of the gut habitat were completely different from those of the other three habitats. Only one core taxon from *Streptococcus* was shared by oral, nasal/pharyngeal and skin habitats.

### Association of Human Oral Saliva Microbes With Environmental Microbes

**FIGURE 5:**
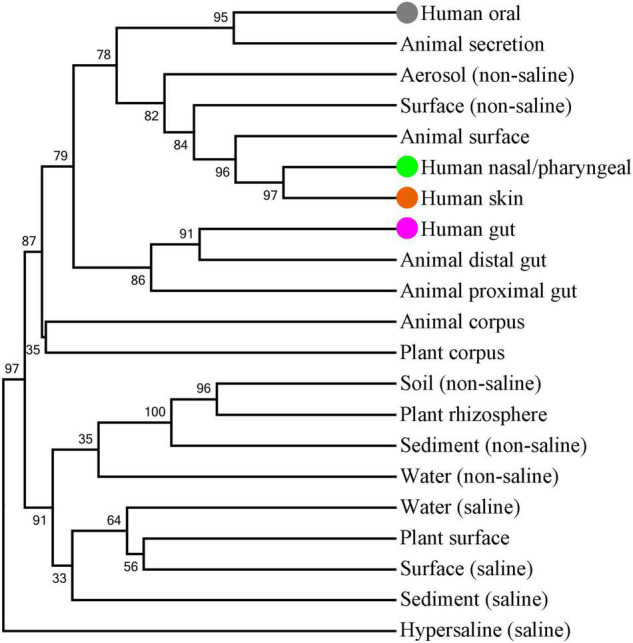
Clustering analysis of microbial composition for human oral saliva, gut, nose/pharynx and skin samples and EMPO environmental labels. The tree was built by using the UPGMA method and Bray-Curtis similarity distance. Bootstrapping with 1,000 re-samplings was performed to determine the robustness of the clustering. The saliva, nose/pharynx, skin and gut are highlighted by gray, light green, orange-red and purple solid spheres, respectively.

A number of microbes are exchanged with the external environment through the human oral cavity. Therefore, we tried to further analyze the association between oral microbes and environmental microbes. EMP classified the samples in different environments into the corresponding environmental labels. These environmental labels were first divided into two categories: Free-living and Host-associated, and further subdivided into 17 subcategories denominated as EMP Ontology (EMPO) level 3. We performed a cluster analysis to display the association of microbe compositions between human oral saliva, gut, nasal/pharyngeal and skin samples and EMPO environmental labels (Figure 5). The results showed that the closest EMPO environmental label to human oral samples was Animal secretion, the closest one to human nasal/pharyngeal and skin samples was the Animal surface, and the closest one to human gut samples was the Animal distal gut. Furthermore, the EMPO environmental labels that were close to human oral samples mostly belonged to the host-associated type but also included the two free-living environments of non-saline Aerosol and Surface. Aerosol and surface are the two types of environments where humans are most in close contact. Specifically, Aerosol is aerosolized dust or liquid. Surface is the biofilm from wet (<5 psu) or dry surface, wood, dust, and microbial mat.

For the 10 genera with more than 1% abundance in the oral microbiome, their abundances in all the EMPO environmental labels were obviously lower than those in the oral cavity. For example, the abundance of *Streptococcus* was 22.62% in the oral cavity, 5.07% in Aerosol (non-saline), 4.22% in Animal surface, 3.02% in Surface (non-saline), 1.35% in Animal proximal gut, and 0.58% in Animal distal gut. Interestingly, all 10 genera had the highest abundance in the Aerosol (non-saline) of the nine free-living environments, as well as the second highest abundance in the Surface (non-saline). Furthermore, we found that the abundance of these 10 genera in various environments had an obviously positive correlation. Therefore, the composition of the oral microbes represented by these 10 genera was specific and had a certain similarity with the microbial composition in the free-living Aerosol and Surface environments.

Aerosol (non-saline) and Surface (non-saline) are the two most closely related types of environments with humans, and the microorganisms in them can be expected to be the most easily transferred to the human body. For different parts of human body, the skin and nasal/pharyngeal microbiomes had the highest similarity with Aerosol (non-saline) and Surface (non-saline) in the microbiome compositions, followed by the oral cavity, and finally the gut. The microbiome compositions of the skin, nose/pharynx, oral cavity, and gut not only overlapped to a large extent but also had a clear gradient from *in vitro* to *in vivo*. On the one hand, the oral cavity communicates microorganisms with the environment in close contact, and on the other hand, oral microorganisms also have a great chance to enter and colonize the intestinal tract along with a large amount of swallowed saliva. These results point toward contact and shared environments being the driving factors of microbial transmission, which is consistent with previous research (Mukherjee et al., 2021). In conclusion, these results emphasize that the oral microbiome is an important link between the environmental microbiome and the human microbiome.

## Materials and Methods

### Human Oral Sample Data Acquisition Based on Earth Microbiome Project Data

The EMP developed a unified standard workflow that leveraged existing sample and data reporting standards to allow biomass and metadata collection across diverse environments on Earth (Thompson et al., 2017). The samples submitted by the global community of microbial ecologists were used to perform the microbiome analysis. DNA extraction and 16S rRNA amplicon sequencing were performed using EMP standard protocols (Caporaso et al., 2011b). The sequence data were error-filtered and trimmed to the length of the shortest sequencing run (90 bp) using Deblur software (Amir et al., 2017).

The EMP data contain a total of 97 studies and 27,742 samples, which are available at http://ftp.microbio.me/emp/release1. We acquired 404 human oral saliva samples from the EMP study to analyze their microbial diversity (Supplementary Data). These oral samples are from 5 independent studies and include the populations from Italy, Puerto Rico, and United States (Caporaso et al., 2011a,b; Piombino et al., 2014). We also selected 216 gut, 253 nasal/pharyngeal and 346 skin samples from the EMP study to proceed with the compared analysis (Caporaso et al., 2011a,b; Lax et al., 2014; Vitaglione et al., 2015).

### Earth Microbiome Project Ontology Classification

The EMP classified the samples in different environments into the corresponding environmental labels (Thompson et al., 2017). The EMPO classified the microbial environments (level 3) as free-living or host-associated (level 1) and saline or non-saline (if free-living) or animal or plant (if host-associated) (level 2). A subset containing 10,000 samples was then generated that gave equal (as possible) representation across environments (EMPO level 3) and across studies within those environments. In this subset, each sample must have ≥5000 observations in the Deblur 90 bp observation table.

### Comparison Against Reference Databases and Core Diversity Analyses

The representative sequences of OTUs were analyzed by the Ribosomal Database Project Classifier algorithm using a confidence threshold of 50% against the Silva 16S rRNA gene database (Quast et al., 2013; Cole et al., 2014).

The alpha diversity was computed with the input Deblur 90 bp BIOM table rarefied to 5000 observations for each sample. The alpha diversity included observed OTUs (number of unique tag sequences), Shannon index (Shannon diversity index), Chao1 index, and Faith’s PD value (Shannon, 1948; Chao, 1984; Faith, 1992).

The clustering of samples was conducted due to storage conditions by PCoA based on Bray-Curtis similarity distance. The Unweighted Pair Group Method with Arithmetic Mean (UPGMA) clustering was based on Bray-Curtis similarity distance. Bootstrapping with 1,000 resamplings was performed to determine the robustness of the clustering. All these analyses were performed with the statistical software PAST (Hammer et al., 2001).

Significant differences in all analyses were evaluated using ANOVA by the software package IBM SPSS Statistics.

### Co-occurrence Network

We selected the 500 most abundant OTUs of the environment types and performed pairwise calculations of the Pearson’s *r* and *p*-values associated with relative abundance using the ‘psych’ package in R. Values of |Pearson’s *r*|>0.5 and *p*<0.05 were considered to indicate valid relationship. The network topological features were calculated using Gephi.

## Data Availability Statement

The original contributions presented in the study are included in the article/Supplementary Material, further inquiries can be directed to the corresponding authors.

## Author Contributions

JLW, JNW, and WC conceived and designed the analysis. JLW, JF, DL, and JNW collected the data and performed the experiments. JLW and JNW performed the analysis. JLW, YZ, JNW, and WC wrote the manuscript. All authors reviewed and agreed with the content of the manuscript.

## Conflict of Interest

The authors declare that the research was conducted in the absence of any commercial or financial relationships that could be construed as a potential conflict of interest.

## Publisher’s Note

All claims expressed in this article are solely those of the authors and do not necessarily represent those of their affiliated organizations, or those of the publisher, the editors and the reviewers. Any product that may be evaluated in this article, or claim that may be made by its manufacturer, is not guaranteed or endorsed by the publisher.

## References

[B1] Amir A. Mcdonald D. Navas-Molina J. A. Kopylova E. Morton J. T. Zech (2017). Deblur rapidly resolves single-nucleotide community sequence patterns. *mSystems* 2:e00191-16. 10.1128/mSystems.00191-16 28289731 PMC5340863

[B2] Atarashi K. Suda W. Luo C. Kawaguchi T. Motoo I. Narushima S. (2017). Ectopic colonization of oral bacteria in the intestine drives TH1 cell induction and inflammation. *Science* 358 359–365. 10.1126/science.aan4526 29051379 PMC5682622

[B3] Bacali C. Vulturar R. Buduru S. Cozma A. Fodor A. Chis A. (2022). Oral microbiome: getting to know and befriend neighbors, a biological approach. *Biomedicines* 10:671. 10.3390/biomedicines10030671 35327473 PMC8945538

[B4] Blod C. Schlichting N. Schulin S. Suttkus A. Peukert N. Stingu C. S. (2018). The oral microbiome-the relevant reservoir for acute pediatric appendicitis? *Int. J. Colorectal Dis.* 33 209–218. 10.1007/s00384-017-2948-8 29273882

[B5] Caporaso J. G. Lauber C. L. Costello E. K. Berg-Lyons D. Gonzalez A. Stombaugh J. (2011a). Moving pictures of the human microbiome. *Genome Biol.* 12:R50. 10.1186/gb-2011-12-5-r50 21624126 PMC3271711

[B6] Caporaso J. G. Lauber C. L. Walters W. A. Berg-Lyons D. Lozupone C. A. Turnbaugh P. J. (2011b). Global patterns of 16S rRNA diversity at a depth of millions of sequences per sample. *Proc. Natl. Acad. Sci. U S A.* 108 (Suppl. 1), 4516–4522. 10.1073/pnas.1000080107 20534432 PMC3063599

[B7] Chao A. (1984). Nonparametric-Estimation of the number of classes in a population. *Scand. J. Statistics* 11 265–270.

[B8] Cole J. R. Wang Q. Fish J. A. Chai B. Mcgarrell D. M. Sun Y. (2014). ribosomal database project: data and tools for high throughput rRNA analysis. *Nucleic Acids Res.* 42 D633–D642. 10.1093/nar/gkt1244 24288368 PMC3965039

[B9] Costello E. K. Lauber C. L. Hamady M. Fierer N. Gordon J. I. Knight R. (2009). Bacterial community variation in human body habitats across space and time. *Science* 326 1694–1697. 10.1126/science.1177486 19892944 PMC3602444

[B10] Dominguez-Bello M. G. Costello E. K. Contreras M. Magris M. Hidalgo G. Fierer N. (2010). Delivery mode shapes the acquisition and structure of the initial microbiota across multiple body habitats in newborns. *Proc. Natl. Acad. Sci. U S A.* 107 11971–11975. 10.1073/pnas.1002601107 20566857 PMC2900693

[B11] Escapa I. F. Chen T. Huang Y. Gajare P. Dewhirst F. E. Lemon K. P. (2018). New insights into human nostril microbiome from the expanded human oral microbiome database (eHOMD): a resource for the microbiome of the human aerodigestive tract. *mSystems* 3:e00187-18. 10.1128/mSystems.00187-18 PMC6280432 30534599

[B12] Faith D. P. (1992). Conservation evaluation and phylogenetic diversity. *Biol. Conserv.* 61 1–10.

[B13] Faust K. Raes J. (2012). Microbial interactions: from networks to models. *Nat. Rev. Microbiol.* 10 538–550. 10.1038/nrmicro2832 22796884

[B14] Gao L. Xu T. Huang G. Jiang S. Gu Y. Chen F. (2018). Oral microbiomes: more and more importance in oral cavity and whole body. *Protein Cell* 9 488–500. 10.1007/s13238-018-0548-1 29736705 PMC5960472

[B15] Gilbert J. A. Jansson J. K. Knight R. (2014). The Earth microbiome project: successes and aspirations. *BMC Biol.* 12:69. 10.1186/s12915-014-0069-1 25184604 PMC4141107

[B16] Gilbert J. A. Meyer F. Antonopoulos D. Balaji P. Brown C. T. Brown C. T. (2010). Meeting report: the terabase metagenomics workshop and the vision of an Earth microbiome project. *Stand. Genomic Sci.* 3 243–248. 10.4056/sigs.1433550 21304727 PMC3035311

[B17] Hammer Ø Harper D. A. Ryan P. D. (2001). PAST: paleontological statistics software package for education and data analysis. *Palaeontol. Electron.* 4:9.

[B18] He J. Li Y. Cao Y. Xue J. Zhou X. (2015). The oral microbiome diversity and its relation to human diseases. *Folia Microbiol. (Praha)* 60 69–80. 10.1007/s12223-014-0342-2 25147055

[B19] Jorth P. Turner K. H. Gumus P. Nizam N. Buduneli N. Whiteley M. (2014). Metatranscriptomics of the human oral microbiome during health and disease. *mBio* 5:e01012-14. 10.1128/mBio.01012-14 24692635 PMC3977359

[B20] Lax S. Smith D. P. Hampton-Marcell J. Owens S. M. Handley K. M. Scott N. M. (2014). Longitudinal analysis of microbial interaction between humans and the indoor environment. *Science* 345 1048–1052. 10.1126/science.1254529 25170151 PMC4337996

[B21] Lira-Junior R. Bostrom E. A. (2018). Oral-gut connection: one step closer to an integrated view of the gastrointestinal tract? *Mucosal Immunol.* 11 316–318. 10.1038/mi.2017.116 29297500

[B22] Mark Welch J. L. Dewhirst F. E. Borisy G. G. (2019). Biogeography of the oral microbiome: the site-specialist hypothesis. *Annu. Rev. Microbiol.* 73 335–358. 10.1146/annurev-micro-090817-062503 31180804 PMC7153577

[B23] Mark Welch J. L. Ramirez-Puebla S. T. Borisy G. G. (2020). Oral microbiome geography: micron-scale habitat and niche. *Cell Host Microbe* 28 160–168. 10.1016/j.chom.2020.07.009 32791109 PMC7604680

[B24] Mukherjee C. Moyer C. O. Steinkamp H. M. Hashmi S. B. Beall C. J. Guo X. (2021). Acquisition of oral microbiota is driven by environment, not host genetics. *Microbiome* 9:54. 10.1186/s40168-020-00986-8 33622378 PMC7903647

[B25] Peters B. A. Wu J. Pei Z. Yang L. Purdue M. P. Freedman N. D. (2017). Oral microbiome composition reflects prospective risk for esophageal Cancers. *Cancer Res.* 77 6777–6787. 10.1158/0008-5472.CAN-17-1296 29196415 PMC5726431

[B26] Philip N. Suneja B. Walsh L. J. (2018). Ecological approaches to dental caries prevention: paradigm shift or shibboleth? *Caries Res.* 52 153–165. 10.1159/000484985 29320767

[B27] Piombino P. Genovese A. Esposito S. Moio L. Cutolo P. P. Chambery A. (2014). Saliva from obese individuals suppresses the release of aroma compounds from wine. *PLoS One* 9:e85611. 10.1371/journal.pone.0085611 24465618 PMC3899019

[B28] Plaza-Diaz J. Ruiz-Ojeda F. J. Gil-Campos M. Gil A. (2018). Immune-Mediated mechanisms of action of probiotics and synbiotics in treating pediatric intestinal diseases. *Nutrients* 10:42. 10.3390/nu10010042 29303974 PMC5793270

[B29] Quast C. Pruesse E. Yilmaz P. Gerken J. Schweer T. Yarza P. (2013). The SILVA ribosomal RNA gene database project: improved data processing and web-based tools. *Nucleic Acids Res.* 41 D590–D596. 10.1093/nar/gks1219 23193283 PMC3531112

[B30] Reddy R. M. Weir W. B. Barnett S. Heiden B. T. Orringer M. B. Lin J. (2018). Increased variance in oral and gastric microbiome correlates with esophagectomy anastomotic leak. *Ann. Thorac. Surg.* 105 865–870. 10.1016/j.athoracsur.2017.08.061 29307454

[B31] Saikaly S. K. Saikaly T. S. Saikaly L. E. (2018). Recurrent aphthous ulceration: a review of potential causes and novel treatments. *J. Dermatolog. Treat.* 29 542–552. 10.1080/09546634.2017.1422079 29278022

[B32] Shannon C. E. (1948). A mathematical theory of communication. *Bell System Tech. J.* 27 379–423.

[B33] Shaw L. Ribeiro A. L. R. Levine A. P. Pontikos N. Balloux F. Segal A. W. (2017). The human salivary microbiome is shaped by shared environment rather than genetics: evidence from a large family of closely related individuals. *mBio* 8:e01237-17. 10.1128/mBio.01237-17 28900019 PMC5596345

[B34] Soffritti I. D’accolti M. Fabbri C. Passaro A. Manfredini R. Zuliani G. (2021). Oral microbiome dysbiosis is associated with symptoms severity and local immune/inflammatory response in COVID-19 patients: a cross-sectional study. *Front. Microbiol.* 12:687513. 10.3389/fmicb.2021.687513 34248910 PMC8261071

[B35] Thompson L. R. Sanders J. G. Mcdonald D. Amir A. Ladau J. Locey K. J. (2017). A communal catalogue reveals Earth’s multiscale microbial diversity. *Nature* 551 457–463. 10.1038/nature24621 29088705 PMC6192678

[B36] Vitaglione P. Mennella I. Ferracane R. Rivellese A. A. Giacco R. Ercolini D. (2015). Whole-grain wheat consumption reduces inflammation in a randomized controlled trial on overweight and obese subjects with unhealthy dietary and lifestyle behaviors: role of polyphenols bound to cereal dietary fiber. *Am. J. Clin. Nutr.* 101 251–261. 10.3945/ajcn.114.088120 25646321

[B37] Wang J. Li D. Wang J. Zhang Z. (2019). Human oral microbiome characterization and its association with environmental microbiome revealed by the Earth Microbiome Project. *bioRxiv [preprint]* 10.1101/732123

[B38] Wasfi R. Abd El-Rahman O. A. Zafer M. M. Ashour H. M. (2018). Probiotic *Lactobacillus sp*. inhibit growth, biofilm formation and gene expression of caries-inducing *Streptococcus mutans*. *J. Cell Mol. Med.* 22 1972–1983. 10.1111/jcmm.13496 29316223 PMC5824418

[B39] Willis J. R. Gonzalez-Torres P. Pittis A. A. Bejarano L. A. Cozzuto L. Andreu-Somavilla N. (2018). Citizen science charts two major “stomatotypes” in the oral microbiome of adolescents and reveals links with habits and drinking water composition. *Microbiome* 6:218. 10.1186/s40168-018-0592-3 30522523 PMC6284318

[B40] Zarco M. F. Vess T. J. Ginsburg G. S. (2012). The oral microbiome in health and disease and the potential impact on personalized dental medicine. *Oral. Dis.* 18 109–120. 10.1111/j.1601-0825.2011.01851.x 21902769

